# Allograft Function as Endpoint for Clinical Trials in Kidney Transplantation

**DOI:** 10.3389/ti.2022.10139

**Published:** 2022-05-20

**Authors:** Luuk Hilbrands, Klemens Budde, Maria Irene Bellini, Fritz Diekmann, Lucrezia Furian, Josep Grinyó, Uwe Heemann, Dennis A. Hesselink, Alexandre Loupy, Rainer Oberbauer, Liset Pengel, Marlies Reinders, Stefan Schneeberger, Maarten Naesens

**Affiliations:** ^1^ Department of Nephrology, Radboud University Medical Center, Nijmegen, Netherlands; ^2^ Department of Nephrology and Medical Intensive Care, Charité Universitätsmedizin Berlin, Berlin, Germany; ^3^ Department of Surgical Sciences, Sapienza University of Rome, Rome, Italy; ^4^ Department of Nephrology and Kidney Transplantation, Vall d’Hebrón University Hospital, Barcelona, Spain; ^5^ Kidney and Pancreas Transplantation Unit, University of Padua, Padua, Italy; ^6^ Department of Clinical Sciences, University of Barcelona, Barcelona, Spain; ^7^ Department of Nephrology, Technical University of Munich, Munich, Germany; ^8^ Department of Internal Medicine, Erasmus MC Transplant Institute, University Medical Center Rotterdam, Rotterdam, Netherlands; ^9^ Paris Translational Research Center for Organ Transplantation, Hôpital Necker, Paris, France; ^10^ Department of Nephrology and Dialysis, Medical University of Vienna, Vienna, Austria; ^11^ Centre for Evidence in Transplantation, Nuffield Department of Surgical Sciences, University of Oxford, Oxford, United Kingdom; ^12^ Department of General, Transplant and Thoracic Surgery, Medical University of Innsbruck, Innsbruck, Austria; ^13^ Department of Microbiology, Immunology and Transplantation, KU Leuven, Leuven, Belgium

**Keywords:** kidney transplantation, graft function, graft dysfunction, clinical study, endpoints

## Abstract

Clinical study endpoints that assess the efficacy of interventions in patients with chronic renal insufficiency can be adopted for use in kidney transplantation trials, given the pathophysiological similarities between both conditions. Kidney dysfunction is reflected in the glomerular filtration rate (GFR), and although a predefined (e.g., 50%) reduction in GFR was recommended as an endpoint by the European Medicines Agency (EMA) in 2016, many other endpoints are also included in clinical trials. End-stage renal disease is strongly associated with a change in estimated (e)GFR, and eGFR trajectories or slopes are increasingly used as endpoints in clinical intervention trials in chronic kidney disease (CKD). Similar approaches could be considered for clinical trials in kidney transplantation, although several factors should be taken into account. The present Consensus Report was developed from documentation produced by the European Society for Organ Transplantation (ESOT) as part of a Broad Scientific Advice request that ESOT submitted to the EMA in 2020. This paper provides a contemporary discussion of primary endpoints used in clinical trials involving CKD, including proteinuria and albuminuria, and evaluates the validity of these concepts as endpoints for clinical trials in kidney transplantation.

## Introduction

As with progressive chronic disease of native kidneys, chronic graft failure results in end-stage renal disease (ESRD) with the need for kidney replacement therapy in the form of dialysis or repeat transplantation. Pathological processes that characterize the late course of graft failure are loss of nephrons, glomerulosclerosis of the remaining nephrons, and interstitial fibrosis and tubular atrophy (1). Essentially, these processes are no different between graft dysfunction and other forms of chronic kidney disease (CKD).

Loss of viable nephrons is reflected in a reduced glomerular filtration rate (GFR), therefore late kidney graft failure is inevitably preceded by a decline in GFR. Notably, the annual rate of eGFR decline in incident dialysis patients with graft failure is higher than in transplant-naïve incident dialysis patients (2): this is potentially related to the hypothesis that multiple factors — or more severe factors — contribute to nephron loss in transplanted patients compared with those who have chronic disease of native kidneys.

The present article provides an overview of primary endpoints used in clinical trials involving CKD, including a contemporary perspective on endpoints for assessing graft dysfunction after kidney transplantation. Biomarkers that have meaningful associations with graft failure are discussed.

## Primary Endpoints for Secondary Prevention of Chronic Renal Insufficiency

The guideline EMA/CHMP/500825/2016 (3) addresses the clinical development of compounds designed to prevent (or slow) processes implicated in chronic renal insufficiency. In its choice of endpoints, EMA distinguishes between primary and secondary prevention of chronic renal insufficiency. In kidney transplant recipients with CKD, the relevant objective is secondary prevention. The recommended primary endpoint in secondary prevention is the time to a predefined loss in GFR, such as a 50% loss. Other (lower) proportions might be used, provided the magnitude is qualified for a specific primary disease or patient population (e.g., extrapolating adult data to pediatric patients). Therefore, three endpoints for graft (dys)function from the EMA 2016 guideline are particularly relevant for kidney transplant recipients (3):

• Kidney function at different timepoints (e.g., 6, 12, and 24 months; 3 and 5 years)
• Proteinuria incidence or worsening
• Time to reach different CKD stages (representing progression of renal damage).

Notably, for primary prevention studies (defined as CKD prevention in patients without any sign of kidney damage), the European Medicines Agency (EMA) recommends using a clinically meaningful and stable GFR loss rate (measured either via slope or time-to-event analyses) as the primary endpoint (3). However, since the EMA guideline was released, additional literature has been published on the choice of CKD endpoints, and various endpoints have been used in clinical trials. There has been increasing use of the eGFR slope in secondary prevention trials, including studies evaluating graft function. These are discussed below.

## Evaluating Graft Function

As initial decline in kidney function is asymptomatic, and clinical manifestations of renal insufficiency occur late in the disease course, general definitions of kidney disease focus on measures of function (e.g., GFR) or damage (e.g., proteinuria, morphological abnormalities). Several CKD biomarkers indicate levels of kidney damage (e.g., active urinary sediment, presence of proteinuria or albuminuria, leakage markers) or functional status (e.g., failure to filtrate plasma or endogenous substances, absorb primary urine, secrete hydrogen ions, or contribute to endocrine function) (3).

Filtration reflects the main function of the kidneys, and GFR is also used as an indicator of kidney function in grafts. Markers for calculating measured (m)GFR must be freely filtered in the glomeruli and not reabsorbed, secreted, or metabolized by renal cells. Although exogenous substances including inulin and iothalamate fulfill these criteria, their analysis requires intravenous infusion; methods to measure their concentrations are costly and are not universally available nor necessarily error free.

## Glomerular Filtration Rate

### Creatinine and Cystatin C

Creatinine and cystatin C measurements are widely used to assess GFR in clinical or research settings, in every relevant patient population (including kidney transplant recipients). Creatinine is a breakdown product of creatine from muscle cells and is largely removed from the blood by glomerular filtration. Therefore, the serum creatinine level, which is easily measured, is a useful reflection of GFR and is traditionally analyzed as an indicator of kidney function. Several decades ago, cystatin C — released by all nucleated cells — was also shown to be a reasonable marker of kidney function. Importantly, however, neither creatinine nor cystatin C meet the requirements of an ideal filtration marker (4). Creatinine levels depend not only on GFR, but also on muscle mass and dietary meat intake; cystatin C levels can also increase with corticosteroid treatment, which is frequently administered after kidney transplantation (5).

Creatinine clearance over a 24-h period can be used as a surrogate marker of GFR. However, creatinine is also secreted, which leads to GFR overestimation. Moreover, 24-h urine collection is burdensome, and inaccuracies in collection cause discrepancies between creatinine clearance and GFR. To overcome such limitations, equations have been developed to calculate eGFR (3, 6, 7). Common denominators in these formulas are serum creatinine and/or cystatin C levels; additional factors used in different combinations are sex, weight, age, and ethnicity. The most frequently used equations derive from the Modification of Diet in Renal Disease (MDRD) study (6) or the CKD-EPI formula (7), which have been validated in transplanted populations (8, 9). However, difficulties associated with measurements based on creatinine and cystatin C levels translate into limitations when applying these formulas, resulting in suboptimal agreement between eGFR and mGFR in the individual patient. Importantly, when it is essential to determine GFR precisely (e.g., when an anticipated decline in function will occur slowly, in longitudinal studies, or when there is considerable variation in non-GFR determinants of biomarkers employed for estimation), the EMA recommends mGFR rather than eGFR (3).

### eGFR Versus mGFR

In kidney transplantation populations, the use of formulas such as MDRD and CKD-EPI to calculate eGFR (8, 9) is hampered by post-transplant differences in body composition (caused by protein catabolic effects of corticosteroids or edema) and inhibition of tubular creatinine secretion by trimethoprim (which is frequently administered). Nevertheless, they are widely used to evaluate GFR in clinical trials of kidney transplantation.

For accurate assessment of kidney function at a given time point in an individual, mGFR is undoubtedly the best available method (10), but this is difficult to undertake in routine practice. However, for comparing cohorts in clinical trial settings, the precise value in the individual may not be required: average eGFR values perform as well in study group comparisons as average mGFR values (11–15). The EMA/CHMP proposes that mGFR is performed in a prespecified subset of patients to confirm eGFR, with creatinine-based eGFR used in preference to cystatin C-based estimations (as creatinine-based eGFR is better characterized). Regardless of the methodology used for eGFR, the influence of confounders on data interpretation should be considered (3).

When selecting an outcome measure of kidney function for clinical research, it is important to know the strength of the relationship between each measure and the occurrence of hard endpoints such as ESRD. No studies show that one-time determination of mGFR is more strongly associated with future ESRD than eGFR, and mGFR has potential limitations, such as the complexity of evaluating large trials. Another major drawback of mGFR evaluation is the impossibility of calculating slopes over time (see below), which requires many repeated measurements.

There is limited agreement between decline in mGFR and eGFR. The Chronic Renal Insufficiency Cohort (CRIC) study compared associations between longitudinal changes (two measurements in 24 months) in eGFR and mGFR (urinary iothalamate clearance) with ESRD risk (16). The strongest association was found for changes in eGFR, which may be explained by higher precision (i.e., less variability) in GFR measurement using eGFR compared with mGFR. In a study of octreotide long-acting release in patients with autosomal-dominant polycystic kidney disease, similar slopes were observed for mGFR and eGFR in intervention and control groups during the 3-year follow-up period (17); comparable studies are not available for the transplant population. In accordance with the prevailing view in the nephrological community, we consider the use of eGFR as a suitable alternative for the practically cumbersome and more costly mGFR, in longitudinal studies and for comparison of investigational groups.

## Proteinuria

Proteinuria is generally measured as the albumin or total protein concentration in a spot sample or in urine collected during a specified time period (e.g., 24 h); in the latter case, the excretion rate of albumin or protein can be calculated. Consequently, EMA/CHMP guidelines state that proteinuria should be assessed quantitatively, using a timed or untimed (spot) urine collection (3). When albumin or protein concentrations are measured in a spot sample, it is important to correct for the urine concentration by simultaneously measuring the creatinine concentration. Accordingly, measurements are expressed as the albumin:creatine ratio (ACR), or protein:creatine ratio (PCR). There is no reason to consider adopting a different policy in kidney transplant recipients.

Since collection of timed urine samples is inconvenient and error prone, use of spot samples has gained popularity. Studies in people with diabetes mellitus, immunoglobulin (Ig) A nephropathy, and a mixed cohort of patients with CKD show that measuring ACR in a morning spot sample is at least equal to measuring 24-h albumin or protein excretion for predicting CKD progression (18–20). In a cohort of 207 kidney transplant recipients, spot and 24-h measurements of albumin and protein excretion were similar predictors of doubling of serum creatinine level and graft loss (21). Therefore, spot sampling can also be recommended in kidney transplant recipients.

Generally, EMA/CHMP guidelines prefer ACR to PCR, especially at low levels of proteinuria, acknowledging that PCR may be the best way to characterize kidney injury (e.g., diabetic nephropathy). Timed urine collection and testing is required after any positive ACR/PCR result to confirm the findings, although repeat ACR/PCR could also be considered. EMA/CHMP guidelines also state that timed urine sample testing would be necessary to assess therapeutic efficacy during a clinical study (3).

## Primary Endpoints of Graft Function in Relation to Chronic Kidney Disease Literature

As mentioned above, progressive decline in kidney graft function in many aspects resembles the course of dysfunction in native kidney disease. However, compared with CKD, it must be realized that after kidney transplantation the course of kidney function is more subject to acute events such as infection and rejection, as well as to changes in immunosuppressive therapy. Literature on kidney endpoints has largely focused on CKD, but data for transplant recipients are available. Data on kidney function endpoints post transplantation were extracted from 213 reports from randomized controlled trials (RCTs) published between 2010 and 2014, comparing immunosuppressive interventions (22). In 44 reports, a measure of kidney function (usually eGFR) was the primary endpoint, although some had other primary endpoints such as graft survival. Table 1 summarizes RCTs in transplantation published after 2014 with kidney function as the primary endpoint (14, 15, 23–30).

**TABLE 1:** RCTs in kidney transplantation with renal function as primary endpoint, published after 2014 (14, 15, 23–30).

Study	Population	Intervention/control	Duration	Renal endpoint	Finding	Comments
APOLLO (23)	*N* = 93; >6 months after Tx sCr <2.5 mg/dl	I: conversion from CNI to EVR	12 months	eGFR: Nankivell	NSD	Premature termination due to slow recruitment. Higher eGFR — MDRD in EVR group
		C: continuation of CNI				
CENTRAL (14)	*N* = 212; 7 weeks after Tx	I: conversion from CsA to EVR	3 years	Change in measured GFR by iohexol or^51^Cr-EDTA clearance from randomization to 36 months	NSD	High rate of study withdrawals. Benefit in renal function in EVR group in on-treatment analysis
		C: continuation of CsA				
SPIESSER(24)	*N* = 145; *dn*Tx	I: SRL	12 months	eGFR: Nankivell	NSD	Benefit in renal function in EVR group in on-treatment analysis
		C: CsA				
Tedesco-Silva et al. (25)	*N* = 256; 90–150 days after Tx	I: conversion from TAC to SRL	24 months	eGFR: MDRD change >5 ml/1.73 m^2^ in on-therapy population (*n* = 195)	NSD	High discontinuation rate in SLR group
		C: continuation of TAC				
Knoll et al. (26)	*N* = 212; >3 months after Tx; eGFR ≥20 ml/min/1.73 m^2^ and proteinuria ≥0.2 g/d	I: ramipril	48 months	Composite endpoint: doubling of sCr, ESRD, or death	NSD	Small numbers per group
		C: placebo				
ELEVATE (27)	*N* = 715; 10–14 weeks after Tx	I: conversion from CNI to EVR	24 months	Change in eGFR — MDRD from randomization to 12 m	NSD	Significantly higher eGFR in EVR group vs CsA subgroup
		C: continuation of CNI				
ADHERE (15)	*N* = 730; 28 days after Tx	I: TAC (8–12 ng/ml until Day 41 and then 6–10 ng/ml) + SRL	12 months	mGFR by iohexol clearance	NSD	High withdrawal rate in the intervention group
		C: continuation of TAC (8–12 ng/ml) + MMF				
3C STUDY (28)	*N* = 394; 6 m after Tx	I: conversion from TAC to SRL	18 months	eGFR—MDRD	NSD	Significantly better renal function in SRL group in on-treatment analysis
		C: continuation of TAC				
BORTEJECT (29)	*N* = 44; presence of DSA and morphologic features of AMR ≥180 days after Tx, eGFR >20 ml/min/1.73 m^2^	I: bortezomib	24 months	Slope of eGFR—Mayo equation	NSD	Small sample size
		C: placebo				
TRANSFORM (30)	*N* = 2037; *dn*Tx	I: EVR + reduced-dose CNI	24 months	Composite of treated BPAR or eGFR—MDRD <50 ml/min/1.73 m^2^ at 12 months	NSD	No difference in eGFR
		C: MPA + standard-dose CNI				

AMR, antibody-mediated rejection; C, control group; CNI, calcineurin inhibitor; CsA, cyclosporine; dn, *de novo*; DSA, donor-specific antibodies; e, estimated; ESRD, end-stage renal disease; EVR, everolimus; GFR, estimated glomerular filtration rate; I, intervention group; m, measured; MDRD, Modification of Diet in Renal Disease; MPA, mycophenolic acid; NSD, no statistical difference; sCR, serum creatinine; SRL, sirolimus; TAC, tacrolimus; Tx, transplantation.

### eGFR as the Endpoint

Doubling of the serum creatinine level is commonly an endpoint in clinical studies of kidney disease, including transplantation: it is considered analogous to a prespecified reduction in eGFR, and is often part of a composite outcome together with initiation of kidney replacement therapy and death from a renal cause (31, 32). However, doubling of serum creatinine is a late event in the progression of kidney insufficiency, and using this measure requires lengthy follow-up and/or very large numbers of patients. Alternative endpoints include the use of less steep (e.g., 30%) declines in eGFR, which have been strongly associated with ESRD (33, 34).

Consistent with findings observed in CKD (33), others have demonstrated that graft failure can be predicted not only by eGFR level at a given time point, but also by decline in eGFR within a relatively short period. For example, data from the Australian and New Zealand Dialysis and Transplant Registry (7,949 transplants) indicated that a ≥30% decline in eGFR between years 1 and 3 post-transplantation was strongly associated with subsequent death-censored graft failure and mortality (35).

The Clinical Trials in Organ Transplantation consortium showed that a 20–40% decline in eGFR at 3–24 or 6–24 months post transplantation was significantly associated with graft loss at 2–5 years, and with absolute eGFR at 5 years (36). The relationship between changes in eGFR and graft loss at 5 years was confirmed in the Genomics of Chronic Renal Allograft Rejection study (36). This suggests that the recommendation of a ≥30% eGFR decline over 2 years as being an acceptable outcome measure in CKD trials could be extended to studies involving kidney transplant recipients.

In some circumstances eGFR may not be a valid surrogate endpoint, although potential solutions for some situations have been formulated (34). For example, in kidney transplantation studies, eGFR decline may not be an ideal surrogate endpoint when drugs that affect muscle mass are administered, such as when muscle atrophy results from corticosteroid treatment (37). In addition, commencing or discontinuing angiotensin-converting enzyme inhibitors, angiotensin receptor blockers (ARBs), or calcineurin inhibitors can acutely affect GFR (38), which could have implications for trial design when eGFR is a surrogate endpoint: inclusion of a run-in period may be warranted.

Several RCTs involving kidney transplant recipients have used eGFR, or a change in eGFR, as a single primary endpoint (23–25, 27, 29, 30, 39). In most cases, eGFR was calculated using the MDRD or Nankivell formulas. Studies compared different immunosuppressive regimens, either *de novo* or starting at a specific time after transplantation. The fact that a significant difference in the primary outcome measure was absent in all but one study is probably not a weakness of the chosen endpoint, but rather illustrates that current immunosuppressive regimens are generally equivalent with respect to short-term graft function.

Therefore, in most circumstances, post-transplantation eGFR seems to be an appropriate endpoint for evaluating graft dysfunction. A systematic review concluded that post-transplantation eGFR (at 12 months) is associated with risk for overall or death-censored graft loss, and all-cause mortality, in univariate and multivariate analyses (40), although such a highly significant association does not necessarily translate into good predictive capability (41). The magnitude of the association between reduced GFR and outcomes was greater for death-censored graft loss versus overall loss, and for graft loss compared with overall patient mortality (40).

### eGFR Trajectories as the Endpoint

Clinical studies in diabetic nephropathy, hypertension and CKD, and polycystic kidney disease use the eGFR slope to evaluate the efficacy of interventions that aim to slow progression of kidney insufficiency (32, 42–45).

In 2017, the KDIGO conference *Challenges in the Conduct of Clinical Trials in Nephrology* concluded that change in eGFR over time was a practical and acceptable method for assessing kidney disease progression (46). KDIGO considered CKD stage and progression rate as determinants of the most useful outcome measure (46). When there is a markedly reduced kidney function (eGFR <45 ml/min/1.73 m^2^) and/or a rapid decline in GFR (>5 ml/min/1.73 m^2^ per year), a composite endpoint consisting of a 30–40% decline in eGFR or the occurrence of ESRD failure is a robust and feasible outcome. When there is a slow decline in kidney function, using the GFR slope as the outcome measure may circumvent the need for lengthy follow-up and/or recruitment of very large numbers of patients. The same considerations can be applied in kidney transplantation studies.

In March 2018, several meta-analyses based on individual patient data were conducted in preparation for the workshop *Change in Albuminuria and GFR as End Points for Clinical Trials in Early Stages of CKD*, which evaluated surrogate endpoints for trials of CKD progression (47, 48). One meta-analysis (14 CKD cohorts) showed that, compared with a rapid decline in eGFR, a slower decline is associated with a lower risk of subsequent ESRD, even in participants with eGFR ≥60 ml/min/1.73 m^2^ (49). A second meta-analysis of 47 RCTs evaluated the GFR slope as a surrogate endpoint for trials examining effects on CKD progression (50). This showed, with sufficiently large sample sizes, that treatment effects on the GFR slope from baseline and 3-month follow-up of 0.5–1.0 ml/min/1.73 m^2^ per year strongly predicted benefits on clinical endpoints such as doubling of serum creatinine, GFR <15 ml/min/1.73 m^2^, or ESRD. Using statistical simulations of GFR trajectories based on data from the 47 RCTs, the GFR slope performed better than clinical endpoints when patients’ initial GFRs were high and not acutely affected by treatment (51). Although cohorts that formed the basis of these meta-analyses did not include kidney transplant recipients, there was a large variation in underlying causes of CKD. This makes it reasonable to assume that the study conclusions would apply to patients with graft dysfunction as a particular form of CKD.

A theoretical advantage of using the eGFR slope as the endpoint, rather than a time-to-event endpoint (e.g., ESRD), is that the decision to initiate dialysis or (re)transplantation can be affected by factors other than GFR. The effect of an intervention on the eGFR slope may therefore better reflect the true effect on kidney graft function.

Importantly, suitability of the eGFR slope as surrogate endpoint depends on patterns of acute and chronic phases of the slope, in the context of the specific disease and potential pharmacodynamic impact of the investigational compound on the slope. The total eGFR slope reflects the slope from time of randomization, i.e., across the entire study period; the chronic slope calculation starts later and is less affected by acute changes in eGFR during the initial phase post randomization. In this respect, transplant recipients likely differ from patients with native CKD. The chance of a non-linear decline in eGFR is probably higher in kidney transplant recipients as a result of acute events such as infection, rejection and initiation or withdrawal of drugs that have acute effects on kidney function, including immunosuppressive agents. This can also impact the number of eGFR measurements needed to calculate the eGFR slope. Such information should be available to evaluate the usefulness of eGFR slope after kidney transplantation and to judge the validity of the chronic slope, versus the total slope. The EMA has indicated that the total slope is generally favored over the chronic slope, because the total slope minimizes possible biases introduced when post-randomization events (e.g., death) or acute changes in eGFR on investigational drug initiation are not considered (48).

The only study in kidney transplant recipients to use the eGFR slope as the primary outcome measure was a clinical trial of bortezomib in late antibody-mediated rejection (AMR) (29). In preparation for a placebo-controlled trial investigating clazakizumab as a treatment for chronic AMR, a data-modeling exercise evaluated the relationship between rate of eGFR decline and risk of graft failure. This was a historical prospective cohort study investigating the relationship between change in eGFR (estimated using the MDRD 4 equation) and risk of graft failure in kidney transplant recipients diagnosed with acute/active (a)AMR (52). The primary analysis used data from 91 patients with biopsy-proven aAMR and baseline eGFR ≥25 ml/min/1.73 m^2^, with a minimum of 3 years’ follow-up data. Both a linear mixed-effects model to describe eGFR decline and a joint model, involving longitudinal eGFR and its rate of decline, were constructed. The joint model predicted that the baseline eGFR and its rate of decline (slope change per month) following an aAMR diagnosis significantly predicts risk of both death-censored and all-cause graft failure. Using the modeling results for all-cause graft loss, the mean eGFR decline from baseline to month 12 after AMR diagnosis was 0.75 ml/min/1.73 m^2^ per month. Using these data, and assuming a 50% reduction in the rate of eGFR decline with clazakizumab, a sample size was calculated for an interim analysis of the 52-week eGFR endpoint. This is an example of how modern statistical techniques can optimize study design in a specific patient population. For these techniques to be used, data are required on the natural course (i.e., without intervention) of kidney function in the population of interest, which should be sufficiently large to accurately define the natural disease course.

### mGFR as a Primary Endpoint

Few RCTs in kidney transplantation have used a change in mGFR as the primary endpoint; mGFR assessment was based on iohexol or ^51^Cr-EDTA clearance (14, 15). Although eGFR values were markedly higher than mGFR values in both studies, the conclusions were not affected when eGFR was used instead of mGFR; thus, no advantage for using mGFR was demonstrated. The BENEFIT and BENEFIT-EXT studies (11, 12) and the Spare The Nephron trial (13) also used mGFR as primary endpoint; the latter illustrated the difficulty in obtaining mGFR data, as no values were available in nine of 112 (8.0%) and 25/116 (21.6%) patients in the mycophenolate mofetil (MMF)/sirolimus and MMF/calcineurin inhibitor (CNI) groups (13). In the BENEFIT study, missing mGFR values were imputed using eGFR values, although the exact magnitude of this imputation is not available (11). Measured GFR is no longer used as a kidney function endpoint in kidney transplantation studies because of limitations mentioned earlier and availability of different methods. In summary, ESOT considers eGFR as the most useful marker for post-transplantation kidney function.

### Proteinuria as the Endpoint

While proteinuria can be considered as a surrogate marker for severity of glomerular damage, proteinuria can also directly contribute to kidney injury and decline in kidney function (53). Although not formally proven, this finding probably also holds true for kidney transplantation populations.

In a large cohort (31,372 individuals from a general population; two or more ACR measurements in 2 years), a fourfold increase in ACR was associated with a threefold heightened risk of ESRD during a median 3 years of follow-up (54). A reduction in proteinuria is also known to protect patients with various forms of renal disease from kidney function decline (55).

In IgA nephropathy, proteinuria is the most widely recognized risk factor for progression to ESRD. Analysis of 13 controlled intervention trials in IgA nephropathy showed an association between treatment effects on percentage reduction of proteinuria, and on a composite of time to doubling of serum creatinine, ESRD, or death (56). Similarly, a meta-analysis of 41 randomized trials in CKD supported use of change in albuminuria level as a surrogate endpoint for CKD progression, particularly in patients with high baseline albuminuria (57). A European Regulator’s perspective on the potential of change in albuminuria as endpoint for clinical trials in CKD has been published (48).

Unlike specific diseases in native kidneys, causes of proteinuria after kidney transplantation are diverse. During the early months after transplantation there may be some contribution from proteinuric native kidneys, but major causes of proteinuria are chronic rejection, recurrence of proteinuric disease, or *de novo* glomerulopathy. Nevertheless, an association between proteinuria and progression to ESRD (demonstrated particularly in diabetes mellitus and IgA nephropathy) has been observed in several cohorts of kidney transplant recipients (58–60). Such findings were confirmed in a *post hoc* analysis of the FAVORIT trial (3,511 participants followed over 4 years), which found that an elevated baseline ACR is independently associated with graft failure, cardiovascular disease, and death (61).

In contrast to studies investigating chronic disease in native kidneys, no studies in kidney transplantation have demonstrated a beneficial effect of proteinuria reduction on progression to ESRD. A clinical trial of ramipril versus placebo in 213 kidney transplant recipients with (mean proteinuria ≥0.2 g/day) showed no difference in the primary outcome (a composite of doubling of serum creatinine, ESRD or death), despite some reduction in mean proteinuria (26). Evidential support for using proteinuria as a post–kidney transplantation endpoint is weaker than evidence for its use in studies of CKD in native kidneys. More evidence is needed from larger cohorts before proteinuria could be proposed for use in kidney transplantation clinical trials.

### Combined eGFR and Proteinuria Endpoint

The KDIGO 2012 guidelines updated the classification system for CKD to include albuminuria, stating that, for the general population, risk of adverse outcomes (mortality, progression to ESRD) at a given eGFR increases with higher levels of albuminuria.

Although studies indicate its promise (26, 61), the combination of eGFR and proteinuria (either as absolute values or as changes from baseline) has not been used as an endpoint in kidney disease clinical trials. Data from the ADVANCE study showed that, in patients with type 2 diabetes mellitus, the 2-year change in eGFR and ACR more strongly predicted the risk of ESRD during a median follow-up of 7.7 years than either of these changes alone (62). A limitation of this study is that the combination of worsening of eGFR and increase in urinary ACR, as well as major kidney events, occurred in only 1% of the study population. Additional studies are required to determine when the combination of changes in proteinuria and eGFR can be used as a surrogate outcome in a broad spectrum of kidney diseases.

The interaction between eGFR and proteinuria — as demonstrated in participants with diabetes in the ADVANCE study — was also observed in kidney transplant recipients. An analysis of linked databases in Canada (*N* = 900) found that rates of death-censored graft loss also increased with lower levels of kidney function at 1 year after transplantation (63). Moreover, within each eGFR category, adjusted rates increased with higher levels of proteinuria. Risk of death-censored graft loss was 49-fold higher for kidney transplant recipients with an eGFR of 15–29 ml/min/1.73 m^2^ and severely increased albuminuria, compared with recipients with an eGFR ≥60 ml/min/1.73 m^2^ and normal protein excretion (62) Although the integration of proteinuria and eGFR assessment has been shown to be a very good predictor of graft outcome (61, 63), more data must be collected before this combination can be advocated as a study endpoint in kidney transplantation clinical trials.

Finally, the causes of long-term graft failure are complex, and there are good arguments to capture this heterogeneity in more integrated composite scoring systems. For this, we refer to the paper in this supplement on surrogate endpoints (64).

## Conclusions

• Chronic renal graft dysfunction resembles CKD of native kidneys in many aspects:
  ○ Loss of nephrons, glomerulosclerosis, interstitial fibrosis, and tubular atrophy are pathological hallmarks of both.
  ○ Dysfunction is reflected as GFR loss, with or without proteinuria, ultimately leading to ESRD; however, ESRD is typically a late event and its use as an endpoint in clinical trials requires very large numbers of patients and prolonged follow-up.
• The EMA 2016 guideline recommended the time to a predefined and justified loss in GFR, such as 50%, as an endpoint in secondary prevention trials.
• Since the guideline was released, additional literature has been published on the choice of endpoints in CKD, and various endpoints have been used in clinical trials.
  ○ Many studies in CKD and kidney transplantation show that a change in eGFR (MDRD or CKD-EPI formulas) is strongly associated with ESRD.
  ○ It is increasingly advocated to use eGFR trajectories as endpoints in intervention trials in CKD. A caveat is the occurrence of an acute treatment effect that hampers use of the GFR trajectory; therefore, in kidney transplantation, special consideration should be given to studies including initiation or discontinuation of calcineurin inhibitors.
  ○ No studies convincingly demonstrate that measured GFR is a better predictor of ESRD than eGFR.
• In studies including patients with advanced-stage graft dysfunction (eGFR <45 ml/min/1.73 m^2^) and/or rapid decline of GFR (>5 ml/min/1.73 m^2^ per year), a composite endpoint consisting of a 30%–40% decline in eGFR or ESRD occurrence is both robust and feasible.
• In studies aimed at improving the lifespan of a transplanted kidney with more conserved renal function (eGFR >45 ml/min/1.73 m^2^), eGFR time course (expressed as slope) should be accepted as surrogate endpoint, provided that the following limitations are considered:
  ○ Use of the chronic eGFR slope is inappropriate when a treatment has acute effects on GFR that are relatively large compared with expected chronic effects. In such cases, use of the total eGFR slope is generally favored.
  ○ Creatinine-based formulas to estimate GFR can be imprecise when there are non-GFR determinants of the creatinine concentration, such as use of drugs that inhibit tubular secretion [trimethoprim] or changes in muscle mass due to corticosteroid treatment.
  ○ Accuracy of cystatin C-based formulas to estimate eGFR can be influenced by corticosteroid use.
• While proteinuria/albuminuria appears to be a useful surrogate endpoint for CKD progression, especially in diabetes and IgA nephropathy, more research must be undertaken before proteinuria (or the combination of eGFR and proteinuria) can be advocated as an endpoint in studies in kidney transplant recipients.

### Scientific Advice From the Committee for Medicinal Products for Human Use (CHMP) of the European Medicines Agency (EMA) Regarding These Conclusions

This paper provides a contemporary discussion of graft functional parameters as primary endpoints in clinical trials as endpoints for clinical trials in kidney transplantation. ESOT has come to the following conclusions:

• The CHMP agreed that endpoints to assess efficacy of medicinal products to slow progression of chronic renal insufficiency (3) can be adopted to trials of kidney transplantation.
  ○ These include hard clinical endpoints (incidence of ESRD and renal/overall survival), proportional decrease in eGFR, and annual decrease in eGFR (slope).
• The CHMP agreed that conceptual approaches used to assess efficacy endpoints for dysfunction can be extrapolated to kidney transplantation, as far as the concomitant medications and diseases are comparable:
  ○ The impact of additional nephrotoxicity (e.g., in cases of CNI or viral nephropathy due to over-immunosuppression) should be delineated from lower potential to preserve functional efficacy.
• The CHMP stated that multiple definitions of efficacy endpoints using GFR have been proposed: the most conservative of these is the 57% reduction in GFR, reflecting doubling of serum creatinine; more recently, lesser degrees of proportional reduction in GFR have been proposed.
• The CHMP agreed that several publications advocate use of eGFR slope as a surrogate for clinical outcome in kidney disease trials, with the following notes:
  ○ eGFR slope should not replace any of the aforementioned GFR-based surrogate endpoints, but should rather be understood as an additional tool to estimate renal benefit; choice of GFR-based endpoint will depend on baseline rate of GFR decline, feasibility issues (e.g., disease prevalence, estimated efficacy of the medicinal product); GFR-based endpoints could also be used to address efficacy in trials of renal transplantation.
  ○ Annualized loss of GFR does not meet all criteria for a valid surrogate endpoint, but (properly defined) is considered as a valuable measure of efficacy in addition to the currently accepted hard clinical endpoints (incidence of ESRD and renal/overall survival). Loss of GFR is most often assessed through serial estimates of GFR (eGFR) but can also be assessed as proportional reduction in GFR (30%–57%).
  ○ The main purpose for a slope-based endpoint in the assessment of therapy in CKD is when feasibility is an issue using standard endpoints, as might be the case in studies of rare and/or early kidney disease. In addition, the value of GFR slope in assessing a medicinal product may be evident during early clinical stages, i.e., in exploratory studies.
  ○ Several issues should be addressed before determining the acceptability of GFR slope as an efficacy assessment in phase III studies to support market access authorization. The main prerequisites are:
    – Low prevalence of the condition as reflected in the target population. It may not be feasible to determine efficacy using standard endpoints in rare diseases or in subpopulations of more common diseases.
    – Slow rate of progression of the kidney disease in the target population. Obviously, assessment of efficacy using standard endpoints may be feasible if rate of progression is rapid.
  ○ Other important considerations:
    – Linearity of the slope. A final decision cannot be made without detailed understanding of nature and patterns of acute and chronic phases of the GFR slope in the context of the specific kidney disease and the pharmacological actions of the investigational compound [see (3, 48)].
    – Suitability of GFR slope as the primary endpoint should be determined on a case-by-case basis. This includes the assessment of how best to analyse efficacy based on eGFR slopes, especially in the context of issues around a possible acute drug effect and linearity assumptions of the GFR measurements.
    – Intercurrent events and confounding. As for any endpoint assessment, development should consider clear definitions of intercurrent events (e.g., death, concomitant medication, treatment discontinuation) and missing data, and a clear understanding of how to handle these issues on a case-by-case basis.
    – Clinically relevant magnitude of effect size. Clinical significance of the proposed difference in slope progressions between treatment arms (active or placebo) should be defined for the specific development. An annual difference of 1 ml/min/1.73 m^2^ for 2 years has been proposed as a clinically significant difference compared with placebo (50). This difference was not accepted as a general cut-off by the CHMP and should be justified for the target population based on baseline GFR and rate of progression of the underlying disease and study population.
    – Efficacy should be supported by other clinical measures, e.g. a second study or other endpoints, most often the standard renal endpoints. The benefit as assessed by these endpoints should be in the same direction as that of the GFR slope.
• The CHMP agreed that proteinuria/albuminuria is of limited value as an endpoint in kidney transplantation.
